# Levels of self-reported and objective physical activity in individuals with age-related macular degeneration

**DOI:** 10.1186/s12889-020-09255-7

**Published:** 2020-07-20

**Authors:** Tjerk Zult, Lee Smith, Charlotte Stringer, Shahina Pardhan

**Affiliations:** 1 Vision and Eye Research Institute, School of Medicine, Faculty of Health, Education, Medicine, and Social Care, Anglia Ruskin University, East Road, Cambridge, CB1 1PT UK; 2 Cambridge Centre for Sport and Exercise Sciences, Department of Life Sciences, Anglia Ruskin University, Cambridge, UK

**Keywords:** Visual impairment, Vision loss, Older adults, GPAQ, Accelerometer, Sedentary behaviour, Physical activity, Exercise

## Abstract

**Background:**

Self-report in people with age-related macular degeneration (AMD) shows that they lead less active lifestyles. Physical activity is important as it has been shown to improve quality of life, reduce co-morbidity and also slow down the progression of AMD. Self-reported measures of physical activity are prone to subjective biases and therefore less accurate in quantifying physical activity. This study compared self-reported and objective (accelerometer-based) physical activity levels and patterns in older adults with AMD.

**Methods:**

Data were collected in 11 AMD subjects with binocular vision loss (aged 76 ± 7 years), 10 AMD subjects with good binocular vision (aged 76 ± 7 years), and 11 controls (aged 70 ± 4 years). Binocular vision was established using visual acuity score. Contrast sensitivity and visual fields were also measured. Self-reported sedentary behaviour and moderate-to-vigorous physical activity (MVPA) was assessed using the Global Physical Activity Questionnaire. Objective measurements were obtained with an Actigraph GT3X accelerometer being worn for seven consecutive days on the hip. The objective physical activity measures were sedentary behaviour, light physical activity, MVPA, and step count.

**Results:**

Objectively measured MVPA was 33–34% higher for controls compared to both AMD groups (*p* < 0.05). There were no group differences for any of the other objectively measured physical activity variables and self-reported physical activity variables were also not significantly different (all *p* > 0.05). Comparing the objective with the self-report physical activity measure showed that all groups under-reported their sedentary behaviour and MVPA, but controls under-reported their MVPA more than both AMD groups (*p* < 0.05). Weak to moderate correlations were observed between the severity of vision loss and objective physical activity measures (all − 0.413 ≥ r ≤ 0.443), while correlations for self-reported physical activity measures were less strong (all − 0.303 ≥ r ≤ 0.114).

**Conclusions:**

People with AMD, irrespective of whether they were vision impaired, were better able to estimate the time spent in MVPA compared to controls. However, objectively measured MVPA, was higher in controls than AMD subjects. Although clinicians may use self-report to monitor the compliance of AMD subjects with any prescribed exercise programs, they should be aware that a valid comparison with healthy controls can only be made when MVPA is objectively measured.

## Background

Physical activity is defined as any bodily movement produced by skeletal muscles that results in energy expenditure [1]. Individuals with vision loss (defined as partial sight or blindness in the better seeing eye) experience various barriers to being physically active [2]. This is of concern as the latest reports from 2010 on global vision loss show that 32 million people were blind and 191 million people had moderate and severe vision impairment [3, 4]. This number is set to double by 2050 because of the ageing population [5]. Age-related macular degeneration (AMD) leads to irreversible loss of central vision and is the second most common cause of blindness and the third main cause of partial sight in high-income countries [6]. The disease mainly affects people aged 50 years and over [7, 8]. The loss of central vision in late AMD results in mobility limitations that make the performance of everyday tasks more difficult [9]. These limitations result in less active lifestyles compared to older adults with normal vision (i.e., increased sedentary time, lower daily step count, and less time spent in moderate-to-vigorous physical activity [MVPA]) [10, 11].

It is important to encourage physical activity because it has been shown to improve quality of life and reduce co-morbidity [12]. In addition, a recent meta-analysis (nine studies from Europe, Australia, and USA; *n* = 66,966) showed that low levels of physical activity were associated with 8 and 41% higher odds of early and late AMD respectively [13]. The authors of this meta-analysis [13] recommended also that a more precise quantification of physical activity in people with AMD is necessary using objective as opposed to self-reported measures. Although self-reported measures have a practical value in monitoring changes in physical activity over time, and in detecting health benefits for large clinical populations, they are more prone to subjective bias and therefore less accurate in quantifying physical activity [14]. To illustrate, self-reported MVPA in prostate cancer survivors has been found to be greatly overestimated [15], and sedentary time was underestimated by 396 min/day in a population of healthy adults [16].

There are several reasons why self-reported and objective measures of physical activity do not correspond. Self-reported measures have been shown to be less accurate in quantifying light-intensity physical activity (e.g., household chores), they are cognitively demanding and are susceptible to recall biases, psychosocial factors such as anxiety, and social desirability of needing to report particular behaviours [14]. It is therefore likely that these biases also exist in individuals with AMD. For example, people with AMD are more likely to have cognitive impairments [17–20] that may affect their cognitive processing of the questions, and accurate recalling of physical activity. The severity of vision loss might also affect self-reported physical activity as AMD subjects with early AMD (good vision) might not feel inclined to report socially desirable behaviour compared to those with late AMD (impaired vision). However, no studies to date have examined how self-reported and objectively measured physical activity compare in individuals with AMD.

The present study compares self-reported and objective (accelerometer-based) physical activity levels in AMD subjects with binocular vision loss, AMD subjects with good binocular vision, and older adults with normal vision. It is expected that older adults with normal vision will be more physically active than AMD subjects without vision loss, with AMD subjects with vision loss being the least physically active. These group-differences will become more pronounced when objectively measured.

## Methods

### Participants

Twenty-three individuals with AMD and 13 older adults with normal or corrected-to-normal vision were recruited to take part in the study. The AMD subjects were divided in a group with vision loss (binocular visual acuity > 0.3 logMAR [21], *n* = 12) and good binocular vision (visual acuity ≤0.3 logMAR [21], *n* = 11). All AMD subjects were diagnosed with AMD (both eyes) by an ophthalmologist. Recruitment of people with AMD took place at local support group meetings and via letters that were sent out by the Macular Society. An age-matched sample of older adults with normal vision was recruited via online advertisements and word of mouth. Inclusion criteria for all subjects were: age ≥ 65 years, live independently (non-institutionalized), and able to walk 100 m without severe physical restrictions. Exclusion criteria were: cognitive impairment, severe neurological or musculoskeletal problems, and eye disorders or ocular pathology affecting eye sight (except AMD). The health of all subjects was assessed through a self-report questionnaire and cognitive function was examined using the Mini Mental State Examination (MSSE). Subjects were considered cognitively impaired when they scored below the required score to pass the MMSE [22]. The last three items of the MMSE require vision but AMD subjects in the present study were able to perform on these items, although some needed help with low vision aids such as a magnifying glass.

All subjects provided written informed consent prior to commencing the study. The study was approved by the Research Ethics Committee of the Anglia Ruskin University (approval number: FMSFREP 16/17008) and is in accordance with the Declaration of Helsinki.

### Study design

Data were collected in a specific time of the year (spring and summer 2018) to minimise the effects of weather and daylight duration on the physical activity patterns between subjects. Subjects visited the laboratory once to do the visual examination, respond to questions of the Global Physical Activity Questionnaire (GPAQ) in a face-to-face interview [23] and receive instructions on the use of the Actigraph GT3X accelerometer (Actigraph Inc., Florida, USA). The questions in the GPAQ were related to a typical week and therefore all subjects were requested to wear the Actigraph GT3X accelerometer during the whole week. All subject started wearing the Actigraph GT3X accelerometer in the first week after the lab visit, and they wore it for seven consecutive days. Once they completed the seven-day wearing period, they returned the Actigraph GT3X accelerometer in person or via mail.

### Visual examination

The visual examination consisted of three tests that were performed with best-corrected spectacles and always in the same order. First, visual acuity was measured binocularly using the Bailey-Lovie logMAR chart at a working distance of 4 m using a letter-by-letter scoring system (0.02 logMAR) [24]. Shorter distances were used when a subject was not able to read the largest size letters at 4 m distance and scores were adjusted accordingly. Second, contrast sensitivity was assessed binocularly using the Pelli-Robson chart at 1 m distance, and scored per group of three letters (0.15 log units) of which two had to be correct [25]. Lastly, visual field examination was conducted monocularly using a Humphrey Field Analyzer (Carl Zeiss Meditec Inc., Dublin, CA) SITA-Standard 30–2 threshold test. The monocular scores were then used to calculate the binocular visual fields using the “best location” model [26].

### Objective assessment of physical activity

Subjects wore the Actigraph GT3X tri-axial accelerometer for seven consecutive days on the right hip. The Actigraph GT3X is an accurate [27] and reliable [28] accelerometer for assessing free-living physical activity. The Actigraph was attached to an elastic belt that was worn around the subject’s waist near the iliac crest. Subjects were instructed to wear the Actigraph during all activities except water-based activities (such as bathing) and when sleeping. The Actigraph measured the frequency, intensity, and duration of physical activity by generating an activity count proportional to the measured acceleration. The time spent at different physical activity intensities was measured over 1-min epochs. The amount of physical activity was expressed as time (min/day) and was classified using the intensity threshold values developed for older adults [29, 30]: sedentary behaviour (0–99 cpm), light-intensity physical activity (100–1040 cpm), and moderate- and vigorous-intensity physical activity (MVPA) (≥ 1041 cpm). Step counts were also recorded by the Actigraph GT3X accelerometer and expressed in steps/day. Subjects needed to have worn the Actigraph GT3X for at least four or more valid days (wear time ≥ 600 min/day) to be included in the analysis. Non-wear time was determined using the criteria defined by Choi et al. [31]. Actigraph data were processed using ActiLife version 6.13.3.

Subjects also filled in an activity monitor log on the days that they were wearing the Actigraph. The typography of the log book was adjusted for people with AMD to increase readability [32]. All subjects were able to read and write in the log book. The activity monitor log served as verification of when and why subjects were not wearing the Actigraph. The Actigraph data matched with the log in all participants.

### Subjective assessment of physical activity

Subjective physical activity levels were assessed in a face-to-face interview using the GPAQ [23]. The GPAQ is developed by the World Health Organisation and consists of 16 questions that examine sedentary behaviour and level of physical activity during work, transport, and leisure time. The GPAQ is a suitable surveillance instrument to monitor physical activity in young and older adults [33, 34]. The level of physical activity is defined as the time spent doing moderate-intensity activities and vigorous-intensity activities. The time spent in moderate-intensity and vigorous intensity were combined and defined as the time spent in MVPA. The experimenter administered the questionnaire and recorded the responses. Data were analysed according to the WHO Steps programme and expressed in min/day [23].

Subjects were also questioned on what types of physical activities they performed during a typical week. These were extracted from the activity scale of the Allied Dunbar Fitness Survey [35] and activities included walking, jogging, swimming, cycling, gardening, stationary biking, tennis, and other aerobic exercises.

### Data analysis

Data in text and figures are expressed as mean ± SD. The statistical analysis was performed using SPSS version 24. Normality was examined using the Kolmogorov-Smirnov test. Each variable was normally distributed. Demographic data were subjected to a univariate analysis of variance (ANOVA) to determine between-group differences. Chi-square test was used to determine between-group differences in gender. Data about the types of exercise were summarized using descriptive statistics. A multivariate ANOVA (MANOVA) was performed to test between-group differences in objective physical activity scores, subjective physical activity scores, and bias scores (i.e., difference score between the objective and subjective physical activity measure). Pillai’s Trace was used to determine between-group effects. A significant MANOVA was followed up by univariate ANOVAs. The variables total wear time, number of valid wear days, and step count were analysed using univariate ANOVA. Bland-Altman analysis [36] were performed to assess the agreement between objective and subjective measures of physical activity for both sedentary behaviour and MVPA. Significant *F* values from the univariate ANOVA’s were subjected to an LSD post hoc pairwise comparison to determine the group means that were different. Pearson’s correlation coefficients were calculated to assess whether there is a relationship between the severity of vision loss and outcomes of the Actigraph and GPAQ. The level of significance (α) was set at *p* < 0.05. Effect sizes were calculated using Cohen’s *d*.

A priory power analysis with G*Power 3.1 was performed to calculate the required sample size to obtain a significant group effect on the MANOVA for bias scores. The effect of AMD on the bias scores has not been examined before. Therefore, a small effect size of 0.20 was used for the power analysis to prevent underestimation of the sample size. The calculated sample size was 11 subjects per group based on an effect size of 0.2 with a power of 80% at the *p* < 0.05 significance level.

## Results

### Subject characteristics

The subject characteristics can be found in Table 1. One AMD subject with binocular vision loss and one AMD subject with good binocular vision were excluded from the analysis because of technical issues with the Actigraph. Two controls with normal vision were also excluded from the analysis because they did not wear the Actigraph for at least three days. Thus, the analysis was performed on data from 11 AMD subjects with vision loss, 10 AMD subjects without vision loss, and 11 controls with normal vision.

**Table 1:** Baseline characteristics of the participants (mean ± SD)

	AMD subjects with vision loss (*n* = 11)	AMD subjects without vision loss (*n* = 10)	Controls with normal vision (n = 11)
Age (years)	76 (7)	76 (7)	70 (4)
Gender (count)			
Male	5	6	5
Female	6	4	6
Mass (kg)	72 (11)	74 (16)	69 (11)
Height (cm)	166 (9)	173 (10)	167 (6)
Visual acuity (logMAR)*	0.73 (0.20)	0.05 (0.12)	0.00 (0.10)
Contrast sensitivity (logCS)*†	0.74 (0.32)	1.37 (0.22)	1.69 (0.07)
Visual fields (dB)*	23 (5)	28 (2)	31 (1)
Mini-Mental State Examination score*	29 (1)	29 (1)	30 (1)

* Significant group difference between AMD subject with vision loss and the other two groups (p < 0.05); † Significant group difference between the AMD subjects without vision loss and controls with normal vision (*p* < 0.05)

### Objective assessment of physical activity

Table 2 shows the data collected with the Actigraph. The univariate ANOVAs revealed that the total wear time, number of valid wear days, and step count were not significantly different between groups (all *p* ≥ 0.173). The MANOVA for the objective physical activity scores showed a significant group effect (*p* = 0.023). Follow-up with univariate ANOVAs showed that there were no significant group differences for sedentary behaviour and light-intensity physical activity (both *p* ≥ 0.103), but there was a significant between-group difference for MVPA (*p* = 0.011). Post hoc analysis revealed significant differences between AMD subjects and controls, in that both AMD groups spent 33–34% less time in MVPA compared to the control group (both *p* ≤ 0.011, d ≥ 1.05).

**Table 2:** Physical activity data measured objectively using the GT3X Actigraph and subjectively using the Global Physical Activity Questionnaire (GPAQ) (mean ± SD)

Variables	AMD subjects with vision loss (*n* = 11)	AMD subjects without vision loss (*n* = 10)	Controls with normal vision (n = 11)	ANOVA *p*-value
Actigraph				
Total wear time (min/day)	838 (75)	873 (61)	880 (62)	0.298
Number of valid wear days	6.9 (0.3)	6.8 (0.4)	6.9 (0.3)	0.710
Sedentary behaviour (min/day)	462 (64)	443 (52)	424 (94)	0.482
Light-intensity activity (min/day)	224 (73)	285 (81)	241 (32)	0.103
MVPA (min/day)	143 (40)*	145 (36)*	216 (87)	0.011
Step count (steps/day)	6218 (3159)	5199 (2529)	8258 (4931)	0.173
GPAQ				
Sedentary behaviour (min/day)	234 (113)	323 (126)	264 (102)	N/A
MVPA (min/day)	102 (78)	127 (92)	92 (50)	N/A
Bias^1^ Actigraph vs. GPAQ				
Sedentary behaviour (min/day)	228 (118)	119 (106)	160 (132)	0.127
MVPA (min/day)	40 (93)*	18 (117)*	124 (86)	0.048

MVPA, moderate-to-vigorous physical activity; N/A, not applicable; ^1^, difference score between the objective and subjective measure of physical activity; *, significantly different from controls with normal vision (p < 0.05)

### Subjective assessment of physical activity

The data of the GPAQ can be found in Table 2. The MANOVA for subjective physical activity scores revealed a non-significant group effect (*p* = 0.114).

**Fig. 1:**
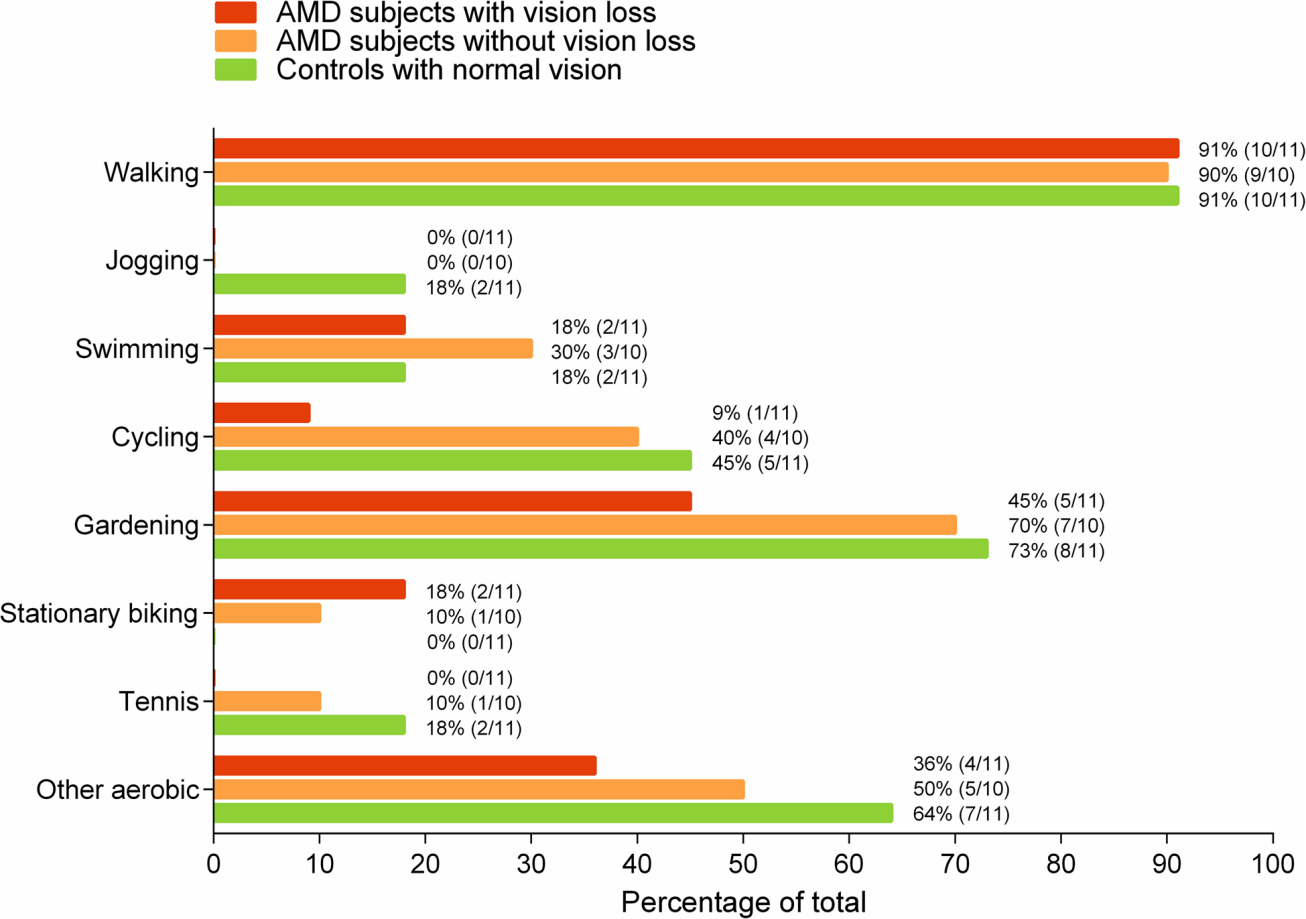
Overview of the exercise types per group. The frequency per group is reported between brackets

Figure 1 shows the reported exercise types that subjects engaged in. Walking and gardening are the most popular forms of exercise among older adults, irrespective of whether they are diagnosed with AMD or not. Cycling seems to be less popular among AMD subjects with vision loss (9%) compared to AMD subjects without vision loss (40%) and age-matched controls with normal vision (45%).

### Objective vs. subjective physical activity measures

**Fig. 2:**
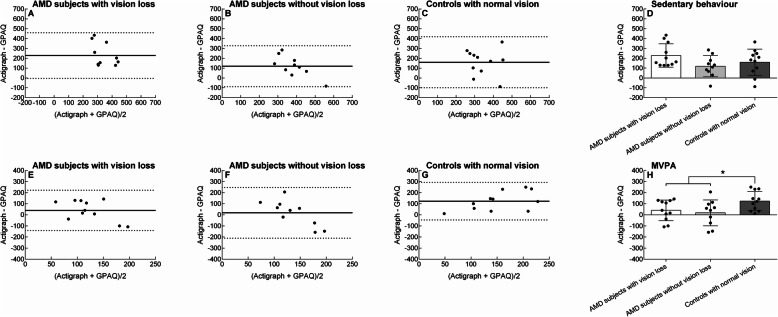
Agreement between the objective (i.e., Actigraph accelerometer) and subjective (i.e., global physical activity questionnaire [GPAQ]) physical activity measure. The panels show the Bland-Altman plots for sedentary behaviour per group (panels **a**, **b**, **c**), difference in agreement between groups (mean ± SD) for sedentary behaviour (**d**), Bland-Altman plots for moderate-to-vigorous physical activity (MVPA) per group (panels **e**, **f**, **g**), and the difference in agreement between groups (mean ± SD) for MVPA (**h**). The round symbols represent individual scores. In Figs. A, B, C, E, F, and G: the solid horizontal line represents the mean difference between the objective and subjective physical activity score (i.e., bias) and the dashed horizontal lines represent the 95% limits of agreement. *, borderline significant difference between groups (*p* ≤ 0.058)

Figure 2A-C demonstrate the results of the Bland-Altman analysis per group for sedentary behaviour. All three groups under-reported their sedentary behaviour compared to the objective measure.

Figure 2E-G illustrate the results of the Bland-Altman analysis per group for MVPA. All three groups under-reported their MVPA compared to the objective measure.

The MANOVA for bias scores showed a group effect (*p* = 0.003). Follow-up of univariate ANOVAs revealed a non-significant group difference for the bias score in sedentary behaviour (*p* = 0.127, Fig. 2D and Table 2) but a significant group difference for the bias score in MVPA (*p* = 0.048, Fig. 2H and Table 2). Post hoc analysis revealed that compared to controls with normal vision, the bias was 68% less for AMD subjects with binocular vision loss (*p* = 0.058, d = 0.94) and 85% less for AMD subjects with good binocular vision (*p* = 0.021, d = 1.04).

### Associations between the severity of vision loss and physical activity

**Fig. 3:**
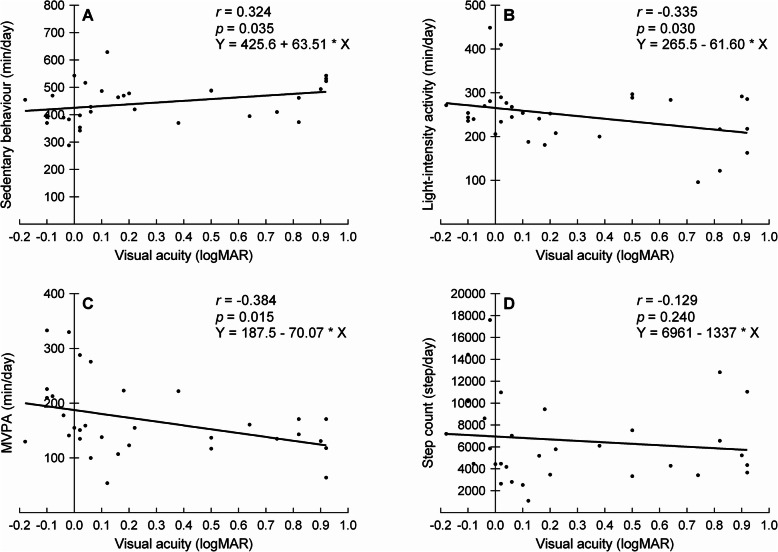
Pearson’s correlations between visual acuity and objective physical activity measures across all subjects. The panels show the correlations for sedentary behaviour (**a**), light-intensity activity (**b**), moderate-to-vigorous physical activity (MVPA) (**c**), and step count (**d**). The round symbols represent individual scores

Table 3 shows that significant associations were found between the amount of vision loss and objectively measured physical activity. Figure 3 illustrates the correlations between visual acuity and the objective physical activity measures. The strength of the significant associations was weak to moderate. No significant associations were found between any of the vision parameters and step count (r ≤ 0.162, *p* ≥ 0.188).

**Table 3:** Pearson’s correlations (r) between physical activity measures and vision loss parameters across all subjects (*n* = 32)

	Visual acuity (logMAR)		Contrast sensitivity (logCS)		Visual fields (dB)	
	R	p-value	R	p-value	r	p-value
Actigraph						
Sedentary behaviour (min/day)	0.324	0.035	−0.384	0.015	−0.413	0.009
Light-intensity activity (min/day)	−0.335	0.030	0.290	0.054	0.150	0.206
MVPA (min/day)	−0.384	0.015	0.443	0.006	0.341	0.028
Step count (steps/day)	−0.129	0.240	0.162	0.188	0.028	0.439
GPAQ						
Sedentary behaviour (min/day)	−0.303	0.046	0.114	0.266	−0.043	0.407
MPVA (min/day)	0.059	0.373	−0.004	0.491	0.055	0.383

GPAQ, Global Physical Activity Questionnaire; MVPA, moderate-to-vigorous physical activity

**Fig. 4:**
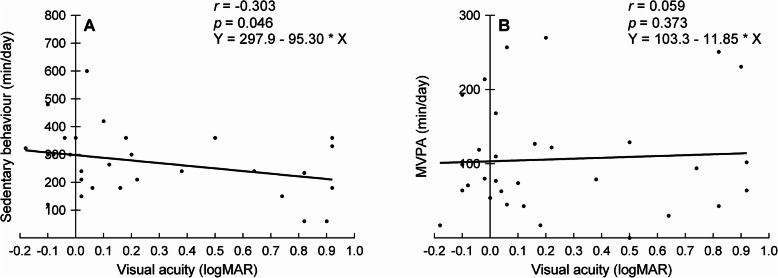
Pearson’s correlations between visual acuity and subjective physical activity measures across all subjects. The panels show the correlations for sedentary behaviour (**a**) and moderate-to-vigorous physical activity (MVPA) (**b**). The round symbols represent individual scores

Table 3 also shows the associations between the amount of vision loss and self-reported physical activity. No significant associations were found except the moderate and significant association between visual acuity and sedentary behaviour. Figure 4 illustrates the correlations between visual acuity and the physical activity parameters of the GPAQ.

## Discussion

The present study is the first to compare self-reported with objective physical activity levels in individuals with AMD. Individuals with AMD, irrespective of whether they were vision impaired, were better able to estimate their MVPA compared to controls. This is in contrast with the hypothesis that the agreement between self-reported and objectively measured MVPA would be less for people with AMD compared to controls. A possible explanation for our findings might be that people with AMD were less physically active than controls when objectively measured and less physically active individuals are known to report their MVPA with less bias [16]. Although clinicians may use self-report to monitor the compliance of AMD subjects with any prescribed exercise programs, they should be aware that a valid comparison with healthy controls can only be made when MVPA is objectively measured.

The agreement between self-reported and objectively measured sedentary behaviour was poor and not significantly different between groups. Compared to the objective measure, sedentary time was on average under-reported 170 min/day which is less than found previously in a sample of British adults (349 min/day) [16]. The difference in under-reporting between the two studies is likely because a more physically active sample of the British population was included in the present study [16].

Compared to controls, AMD subjects with and without vision loss engaged 71–73 min/day less time in MVPA when objectively measured, but between-group differences were absent with self-reported MVPA data. Previous accelerometer-based research showed that individuals with vision loss were more sedentary and less active in both light-intensity and MVPA [11]. However, and in contrast to the findings of the present study, physical activity levels in AMD subjects with good binocular vision were not significantly different from healthy controls [11]. It is hard to compare their findings with ours because they used different criteria to measure light-intensity activity (100–2019 cpm vs. 100–1040 cpm) and MVPA (≥ 2020 cpm vs. ≥ 1041 cpm). The criteria in the present study were chosen because they are specifically developed for older adults [29]. Although our people with AMD spent less time in MVPA compared to controls, they met the physical activity guidelines of being moderately active for at least 150 min per week [37].

The number of steps per day was not different for AMD subjects with and without good binocular vision compared to healthy controls. However, AMD subjects with and without good binocular vision did not achieve the recommended 7000–10,000 steps/day [38] in contrast to the healthy controls who walked 8258 steps/day. Walking is the primary form of MVPA in older adults but older adults with AMD seem to engage less in other types of MVPA (see Fig. 1). The reasons that older adults with AMD engage less in other physical activities are likely related to the environment (e.g., transportation difficulties, lack of accessible exercise equipment) and to the societal attitude towards people with vision loss [2].

There was a weak to moderate relationship between the severity of vision loss and any of the objectively measured physical activity levels. These findings support previous research where a loss in visual acuity, contrast sensitivity, and visual field were all associated with less engagement in MVPA [11, 39]. The present study is the first to show that vision loss is associated with less engagement in light-intensity activities and more sedentary behaviour. Interestingly, correlations between vision loss and self-reported physical activity levels were less strong and in the opposite direction than when physical activity levels were objectively measured. Objective instead of self-report measures of physical activity should be used in future research to accurately quantify associations between vision loss and physical activity.

Limitations of hip-worn accelerometers are that they cannot be worn during water-based activities and that they underestimate the activity count of lower limb movements (e.g., cycling). Ten of our participants used a bicycle for transportation (1 AMD subjects with vision loss, 4 AMD subjects without vision loss, 5 controls) and seven subjects participated in swimming (2 AMD subjects with vision loss, 3 AMD subjects without vision loss, 2 controls) so it is likely that our objectively measured physical activity levels are underestimated. Therefore, the discrepancy between self-reported and accelerometer-based MVPA might have been more than presented in each group.

The difference in measurement timeframe between administering the GPAQ and the accelerometer might also have affected the results. The GPAQ is developed to measure physical activity during “a typical week” and was administered before subjects started to wear the accelerometer. The accelerometer was worn during “a typical week” but wearing an accelerometer and using a log might have resulted in subjects becoming more physically active than usual (i.e., Hawthorne effect). Had the GPAQ been administered after the subjects completed the wearing of the accelerometer, GPAQ responses might have been a better reflection of “a typical week” in which the accelerometer was worn. The effect of administration time on self-reported physical activity will be similar across groups and will therefore not affect the conclusions of the present study.

## Conclusions

In this study, all the subjects under-reported their sedentary behaviour and MVPA. The agreement between self-reported and objectively measured MVPA was better in AMD subjects with and without vision loss than controls. Thus, in contrast to our hypothesis, individuals with AMD did not have a tendency to self-report socially desirable behaviour. However, people with AMD, irrespective of whether they were vision impaired, engaged less in MVPA than controls when objectively measured and these between-group differences would have remained unnoticed had MVPA only been measured subjectively. Clinicians may use self-reported physical activity levels to monitor the compliance of AMD subjects with prescribed exercise programs but they should be aware that a valid comparison with healthy controls can only be made when MVPA is objectively measured.

## Data Availability

The datasets used and analyzed during the current study are available from the corresponding author on reasonable request.

## Abbreviations

AMD: Age-related macular degeneration
ANOVA: Analysis of variance
GPAQ: Global Physical Activity Questionnaire
MANOVA: Multivariate analysis of variance
MSSE: Mini Mental State Examination
MVPA: Moderate-to-vigorous physical activity
